# Next-Generation Probiotics: Microflora Intervention to Human Diseases

**DOI:** 10.1155/2022/5633403

**Published:** 2022-11-16

**Authors:** Huanchang Zhang, Yunfeng Duan, Feng Cai, Demin Cao, Lei Wang, Zhenyi Qiao, Qing Hong, Nan Li, Yuanrong Zheng, Miya Su, Zhenmin Liu, Baoli Zhu

**Affiliations:** ^1^State Key Laboratory of Dairy Biotechnology, Shanghai Engineering Research Center of Dairy Biotechnology, Dairy Research Institute, Bright Dairy & Food Co., Ltd., Shanghai, China; ^2^Institute of Microbiology, Chinese Academy of Sciences, Beijing, China

## Abstract

With the development of human genome sequencing and techniques such as intestinal microbial culture and fecal microbial transplantation, newly discovered microorganisms have been isolated, cultured, and researched. Consequently, many beneficial probiotics have emerged as next-generation probiotics (NGPs). Currently, “safety,” “individualized treatment,” and “internal interaction within the flora” are requirements of a potential NGPs. Furthermore, in the complex ecosystem of humans and microbes, it is challenging to identify the relationship between specific strains, specific flora, and hosts to warrant a therapeutic intervention in case of a disease. Thus, this review focuses on the progress made in NGPs and human health research by elucidating the limitations of traditional probiotics; summarizing the functions and strengths of *Akkermansia muciniphila*, *Faecalibacterium prausnitzii*, *Bacteroides fragilis*, *Eubacterium hallii*, and *Roseburia* spp. as NGPs; and determining the role of their intervention in treatment of certain diseases. Finally, we aim to provide a reference for developing new probiotics in the future.

## 1. Introduction

Over the past 15 years, developments in the field of microbiome research have reshaped our view of human microbiology. We can now accurately describe the composition and function of microflora from different parts of the body and associate them with potential diseases, risks, and obvious clinical symptoms. The intestinal tract is an important part of the human body, and regulation of intestinal microflora is a key factor in determining host metabolism to suggest potential therapeutic modalities in case of a diseased condition. For instance, probiotics and fecal microbiota transplantation (FMT) are considered as promising solutions.

Probiotics are defined as “living microorganisms, when given in sufficient amounts, are good for the health of the host” [1]. Probiotics help the intestinal tract in returning it to its original pre-maladjusted state or by creating a new microflora balance [2], thereby eliminating or alleviating underlying diseases. However, the clinical efficacy of studies on probiotics and the mechanism of flora intervention are both unclear. With the emergence of FMT, biological therapeutic strategies have been developed and successfully applied for treating diseases such as *Clostridium difficile* infection (CDI) [3] and Crohn's disease [4]. Although FMT is effective, its long-term effects have not been fully studied. Thus, which microorganisms should be transplanted, which strains are functional, and how many strains are needed to effectively change the intestinal flora are still important unanswered questions [5].

**Figure 1:**
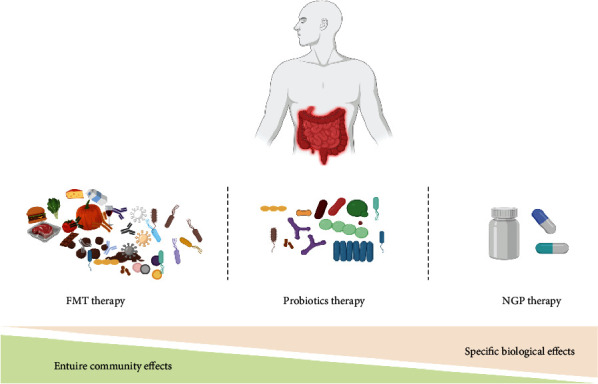
Moving from microflora intervention to human diseases. The important contribution of the gut microbiota to human health and disease is well-recognized and described. FMT can be a collective ecosystem, which alter the overall microbial composition of the gut via altering the overall microbial composition of the gut. FMT therapy could be affected by transmissibility of disease-causing agents and resilience of preexisting microbial community. Conventional probiotics are effective and low cost but may have some limited health benefits. Due to limitation in the use of conventional probiotics, NGPs represent a promising class of biologics for live biotherapeutics.

Rapid development of high-throughput sequencing technology and bioinformatics has deepened our understanding of the relationship between human microbiome [6], intestinal microbiome, and human diseases. As a result, many gut microbes that are difficult to culture in vitro have been isolated and identified [7]. Many strains with potential health benefits are gradually emerging. These potential strains can be used as candidates for next-generation probiotics (NGPs) and can be used as active biological agents in clinical settings for targeted treatment of certain diseases [8] (Figure 1). However, the exact mechanism of interaction of NGPs with the host is undetermined. Here, we summarize and provide the latest information on NGPs with respect to the physiological characteristics, safety, pathogenicity, drug resistance, and their effects on host health and diseases. Further, we aimed to explore the mechanism between NGPs and human diseases to find candidate NGPs for clinical applications.

## 2. Interaction between Microbiota and Host Intestinal Health

Human intestinal microflora is established at birth, and its diversity gradually matures before the age of three with the development of environmental and dietary patterns [9]. The intestinal microflora of adults is composed of approximately 39 trillion microbial cells, which is equivalent to the number of human cells [10, 11]. Ideally, the human host lives in harmony with its complex intestinal microflora and is in a state of intestinal balance.

However, because of drug usage, infections, aging, lifestyle, surgery, and malnutrition, the intestinal flora may become imbalanced, thereby leading to a series of acute or chronic diseases such as inflammatory bowel disease (IBD), irritable bowel syndrome (IBS), cirrhosis, nonalcoholic fatty liver disease, and gastrointestinal malignancies. Therefore, intestinal microbiota may play a key role in human diseases, and flora intervention may be a potential treatment option. In a study on intestinal flora conducted by Bäckhed et al., researchers transferred bacteria from the cecum of obese mice to aseptic mice and found that the aseptic mice showed symptoms of obesity [10]. Further experiments found that the phenotype of obesity could also be transferred from people with obesity to aseptic mice [12], thereby initiating studies on FMT intervention.

### 2.1. Microbiota Transplantation Indicates a Causal Role in Human Disease

The FMT process involves taking stool samples from healthy donors and administering them to a patient through various routes such as mouth or rectum. In fact, the idea of using a mixture of fecal bacteria to treat intestinal diseases has been around for centuries; however, it was not until 1958 that the first case of FMT to treat pseudomembranous colitis caused by C. difficile was officially reported [13]. Clinical data show that FMT is very effective as the primary treatment of CDI [13]. In the USA, FMT is performed at least 10,000 times a year [14], and more than 200 clinical studies have been conducted worldwide. At present, this method is being used in the treatment of complex diseases such as IBD, metabolic diseases, and autism [15].

However, FMT is a double-edged sword. Donor feces are complex biological samples, differ from individual to individual, and are accompanied by the risk of introducing disease-causing microorganisms that may ultimately worsen the disease [16]. For instance, two immunocompromised patients developed drug-resistant *Escherichia coli* bacteremia after receiving FMT capsules from the same donor, independently, and one patient died [14]. Consequently, the US Food and Drug Administration (FDA) has issued a safety warning regarding the use of FMT, wherein they suggest that it is necessary to screen and test the donor for multidrug-resistant bacteria to reduce the transmission and infection of drug-resistant bacteria during experimental FMT. Obviously, in addition to conducting a unified screening survey of FMT donors to reduce risk, there is also a need to improve FMT or suggest alternative drugs for thousands of patients who develop recurrent CDI each year. Therefore, scientists must determine which donor feces samples are beneficial. Although this is a major challenge for FMT research, it presents an opportunity to develop robust and medically precise treatment regimens to improve the patient health.

### 2.2. Why Are NGPs Essential?

Probiotics are effective regulators of intestinal microflora balance [17]. They have a long history of safe consumption with a range of health benefits, such as preventing neonatal necrotizing enterocolitis (NEC) [18], reducing the frequency of crying in infants with intestinal colic [19], improving the quality of life in patients with IBS [20], preventing diarrhea in children's hospitals [21], and reversing intestinal inflammation caused by antibiotics [22]. Enthusiasm and popularity within the community for probiotics have led to a multibillion-dollar industry worldwide [23], which is expected to exceed $65.9 billion by 2024 [24]. Probiotics market size, share & trends analysis report, by ingredient type (bacteria and yeast), by form (liquid probiotic and dry probiotic), by application (food & beverages, dietary supplements, and animal feed), by end user (human probiotics and animal probiotics): global industry perspective, comprehensive analysis, and forecast, 2019–2026. http://www.zionmarketresearch.com/report/probiotics-market. Accessed 4 July 2021). Most of the probiotics on the market are from the conventional *Lactobacillus*, *Streptococcus*, *Bifidobacterium*, yeast, and *Bacillus* [25]. For instance, *β*-galactosidase secreted by *S. thermophilus* in yogurt can break down lactose into digestible glucose and galactose, which is beneficial for those with lactose intolerance. However, probiotics are not considered drugs in many countries and are largely unregulated [26]. As a result, a mixture of probiotic products is usually marketed directly to consumers without a reliable proof of efficacy [22]. On investigating the views of 208 general practitioners and 207 medical experts in the Netherlands, only 51% of the experts recommended the use of probiotics [27], as although many strains aid in disease prevention, their clinical efficacy is unreliable [28]. NEC is a destructive intestinal disease with a high incidence rate in premature infants and is characterized by eating intolerance, abdominal distension, bloody stool, high mortality, and unknown etiology [29]. Previous meta-analyses have found that probiotic supplements reduce the incidence of NEC in very low birth weight newborns (<1500 g) from 6% to 2%, even if *Lactobacillus* and *Bifidobacterium* are used alone [30]. However, results of a phase III clinical trial have shown that *Bifidobacterium* brevis BBG-001 has no significant effect on the prevention of NEC and late-onset septicemia in premature infants [31]. Importantly, in 89 studies related to acute infectious gastroenteritis, 58 reports included only the duration of diarrhea as an experimental outcome. Consequently, there is insufficient evidence of *Saccharomyces cerevisiae boulardii*, *L. rhamnosus* ATCC 53103, *L. rhamnose* ATCC 53103, *L. rhamnose* R0011, and *L. helveticus* R0052 as probiotics [32]. In fact, the American Gastroenterological Association has recommended that probiotics should not be used in children with acute infectious gastroenteritis in the USA and Canada [32]. Thus, even though probiotics are promising, the existing research that supports their claims is variable, and very few clinical verifications have been conducted. Further, all individuals have a unique intestinal microflora; thus, the effects of different bacteria on different people are likely to be variable as well. Therefore, further investigations to determine the possible effects of probiotics are warranted in future. The effects of traditional probiotics on human intestinal microbiota are limited, and the efficacy evaluation of flora intervention should be based on strain level. Approximately 80% of intestinal bacteria are unknown [33], and more than 25 types of immune metabolic diseases are related to flora abundance in the body. Specific species show better resilience to inflammation, tumors, and metabolic diseases [34, 35]. However, very few studies have been conducted on them as these bacteria are extremely sensitive to oxygen, and it is difficult to obtain their pure cultures.

**Figure 2:**
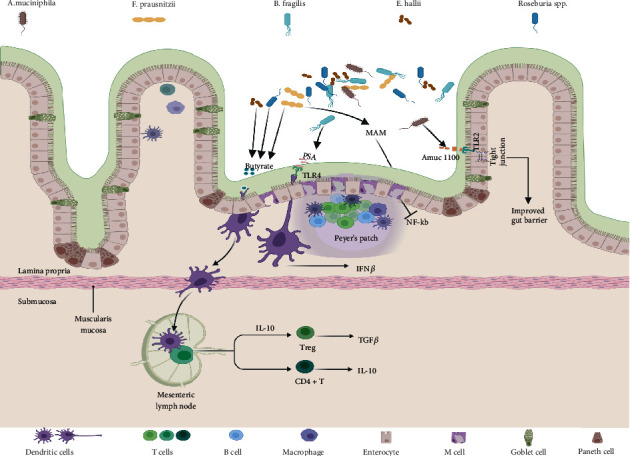
Major mechanisms involved in the crosstalk between NGPs and host. This illustration contains 5 types of intestinal microbiota and their changes mentioned in this review. Each of these mechanisms may play a role in the onset and progression of many inflammatory diseases, although the precise mechanisms are not known. The mechanisms underlying the effects of certain microbiota on the gastrointestinal are described. Damage and inflammation at the intestinal barrier can result in a break in self-tolerance and potential for increased systemic inflammation. MAM inhibited the activation of NF-kappa B pathway, and Amuc 1100 derived from *A. muciniphila* metabolite are known to support intestinal barrier integrity, by increasing expression and organization of tight-junction proteins. Another important class of microbiota acting on DC cells by secreting substances such as PSA and butyrate. Then, DC cells further act on CD4+ T cells or regulatory T (Treg) cells to inhibit inflammation.

With the development of culturomics, pure cultures of bacteria such as *Akkermansia muciniphila*, *Faecalibacterium prausnitzii*, *Bacteroides fragilis*, *Eubacterium hallii*, and *Roseburia* spp. have successfully been obtained in vitro. These have been termed as NGPs as they expand the understanding of underlying mechanisms of probiotic-host health interactions [36, 37]. In 2017, the Nature Microbiology formally suggested the concept of NGPs for the first time [8]. They believe that NGPs differ from traditional probiotics and adhere to the attributes of “active biological agents” as per the US FDA guidelines: (1) contain live organisms, such as bacteria; (2) are applicable for prevention, treatment, or cure of a disease or condition in human beings; and (3) are not a vaccine. The NGPs should undergo clinical trials (phases I–IV) and must be approved by relevant regulatory authorities before being put on the market [8]. Nowadays, candidate strains of NGPs are being widely studied and reported, Each of these mechanisms may play a role in the onset and progression of many inflammatory diseases, although many of these are still at the very early stage of mechanistic investigation (summarized in Figure 2). NGPs reportedly overcome the shortcomings of existing probiotic preparations and can play an important role in the intervention or treatment of human diseases in the future [38]. In the following sections, we will discuss some potential NGP candidates.

### 2.3. NGP Microorganisms Candidates

#### 2.3.1. Akkermansia muciniphila

*Akkermansia muciniphila* is a common, elliptic, gram-negative bacterium that belongs to the Akkermansiaceae family and is one of the strongest candidates for NGPs. In humans, this bacterium is symbiotic, and in healthy people, the abundance of *A*. *muciniphila* is greater than 1%–5% [39]. Mucus is composed of glycoproteins that are produced and degraded in the colon and are known as a “prebiotic” in the human body. In 2004, Derrien et al. isolated and identified a new mucin-degrading bacterium through a combination of different analytical techniques (denaturing gradient gel electrophoresis, most probable number approach, and soft agar technique) [39]. This bacterium colonizes the outer mucin layer of host intestinal epithelial cells and degrades intestinal epithelial mucin exclusively. It also stimulates intestinal epithelial cells to produce new mucin proteins; increases intestinal mucin layer thickness; promotes the release of Foxp3^+^, Treg, interleukin-10, and tumor growth factor-*β*; reduces islet inflammation; and slows the development of type 1 diabetes [40].

*A. muciniphila* steadily colonizes the human intestinal tract during the first year of life to eventually reach the same level as that in healthy adults. Importantly, compared with healthy people, patients with obesity, type 2 diabetes (T2D), IBD, high blood pressure, or liver diseases have a lower fecal flora abundance of *A. muciniphila*. Although *A. muciniphila* can effectively restore the efficacy of antitumor drug PD-1 [41], most studies have not attempted to elucidate its causal relationship with a disease. Everard et al. [42] have shown that obese mice have a lower abundance of *A. muciniphila*. Thus, *A. muciniphila* can reverse obesity induced by a high-fat diet, promote “passivation” of metabolic endotoxins, promote release of inflammatory bacterial lipopolysaccharides, and effectively reduce insulin resistance and heart metabolic complications [43]. Plovier et al. [44] have found that the treatment of *A. muciniphila* under pasteurized conditions at 70°C for 30 min significantly increases colonic length and depth and increases resistance to obesity and insulin resistance; this mechanism is closely associated with the recombinant protein Amuc_1100^∗^. Ottman et al. [45] have confirmed through genomic and proteomic analyses that the outer membrane of *A. muciniphila* is rich in proteins encoded by a specific type IV pili gene cluster. Furthermore, the 32 kDa Amuc_1100 protein^∗^ is the most abundant here [46] and very stable at 70°C. Further experiments have shown that this recombinant protein can signal toll-like receptor 2 expression in cells just like *A. muciniphila* does, reduce body fat accumulation, and improve intestinal barrier function [44]. Moreover, unlike living *A. muciniphila*, inactivated *A. muciniphila* do not stimulate energy consumption through the Browning effect of white fat cells, but do so by reducing lipid drops adjustment factor perilipin 2-related proteins, increasing energy discharge [47], increasing the number of intestinal goblet cells (responsible for the production of mucus), reducing the fat cell diameter, and reducing the ability of the host to obtain energy from diet [44]. Therefore, Amuc_1100^∗^ is a potential probiotic for preventing and treating colitis. Inactivated *A. muciniphila* may also be utilized for the prevention and treatment of diseases.

The potential pathogenicity of *A. muciniphila* is mainly by its adherence to the intestinal mucous layer to degrade mucosal proteins; however, to date, no studies have explored the association between increased abundance of *A. muciniphila* and disease mechanisms. After the development of a synthetic medium for large-scale enrichment of *A. muciniphila*, Plovier et al. have conducted experiments in animals [44] and humans [48] to evaluate the safety and tolerance of *A. muciniphila* in 40 participants with insulin resistance for the first time. They observed that after the participants consumed 1 × 10^10^ CFU/mL *A. muciniphila* every day for three months, the benefits of supplementing living bacteria were not significant, and the dead bacteria significantly decreased insulin levels, insulin resistance, total plasma cholesterol, white blood cell count, and blood fat polysaccharides. An average weight loss of 2.3 kilograms was accompanied by a slight reduction in fat mass and hip circumference, both of which are safe and well-tolerated, and do not affect the overall intestinal flora [48]. The Pendulum Therapeutics markets biostime capsule products such as “Pendulum Glucose Control” that have *A. muciniphila* WB-STR-0001 in them to control T2D development [49]. Revolutionizing metabolic health and type 2 diabetes management. https://pendulumlife.co-m/. Accessed 4 July 2021). Therefore, we suggest that it is safe to use *A. muciniphila* in a reasonable dose and strictly limit its use to an applicable population. We have previously determined that the fermentation products of Paenibacillus bovis sp. nov. BD3526 can alleviate the symptoms of T2D in Goto-Kakizaki Rats by increasing the abundance of *A. muciniphila* [50].

#### 2.3.2. Faecalibacterium prausnitzii

*Faecalibacterium prausnitzii* belongs to the Firmicutes phylum and *Clostridium leptum* group cluster IV. It is frequently described as a gram-positive, long-rod-shaped bacterium that is present in the intestinal tract of healthy adults and accounts for approximately 5% of the total fecal microflora [51]. Indeed, the depletion of *F. prausnitzii* in the gut microbial community has been associated with microbial dysbiosis coincident with a broad range of metabolic and/or immune-mediated chronic diseases, such as T2D [52], inflammatory diseases [53], and obesity [54]. Unlike *A. muciniphila*, *F. prausnitzii* cannot utilize intestinal epithelial mucin; however, studies show that mucin can improve the abundance of this bacterium [55]. High abundance of *F. prausnitzii* can stimulate the resynthesis of mucin and tight junction proteins and can repair damaged intestinal mucosal barrier [56]. *F. prausnitzii*, along with *B. thetaiotaomicron*, can regulate the differentiation of intestinal goblet cells, mucus secretion, and glycolylation and maintain mucus barrier homeostasis [57]. Therefore, it is often used as the preferred biomarker for identifying adolescents with obesity [58] and ulcerative colitis.

*F. prausnitzii* A2-165 promotes ovalbumin-specific T cell proliferation, reduces the number of interferon-*γ*^+^ T cells, and demonstrates a strong ability to induce human and mouse dendritic cells to secrete IL-10 [59]; thus, these studies stimulate interest in *F. prausnitzii* as an NGP. In individuals with obesity, the fat cells swell, which lead to local hypoxia of tissues, cell death, leukocyte infiltration, and systemic inflammation [60]. *F. prausnitzii* ATCC 27766-treated mice showed reduced CD45^+^ inflammatory degree (*P* = 0.006), increased adiponectin expression in visceral adipose tissues, and reduced inflammation in adipose tissues [61]. However, this bacterium is extremely sensitive to oxygen, and strict culture conditions limit its study and application. Therefore, researchers have attempted to replace the live bacteria with the supernatant cultured by *Faecalibacterium prausnitzii* for many studies.

*F. prausnitzii* ferments glucose to produce short-chain fatty acid butyrate, formate, a small quantity of D-lactic acid [62], medium-chain fatty acids, and salicylic acid [63]. Butyrate provides an energy source for epithelial cells, regulates intestinal T cell activity, induces colon cancer cell apoptosis, inhibits intestinal inflammation, and improves metabolic syndrome [64]. Salicylic acid, which is an anti-inflammatory compound, can promote the differentiation of immune cells through an anti-inflammatory process and maintain the immune homeostasis of the body [59]. Importantly, in *F. prausnitzii* culture supernatant, researchers have isolated microbial anti-inflammatory molecule (MAM), which has anti-inflammatory effects, MAM administration inhibited NF-*κ*B and reduced the production of the proinflammatory mediator-like Th1 cytokines, and Th17 cytokines in the colon [65]. In addition, the supernatant of *F. prausnitzi*i SPAH strain activates the expression of collagen genes *ACTA2* and *Col1a2* and have protective effects on skin wound inflammation and [66]. Currently, *F. prausnitzii* isolated from healthy populations has shown good anti-inflammatory properties in vitro [67]. Although supporting evidence of its safety has been proven in a study in calves [68], its potential role and safety in human pathogenesis are unclear. Genome-wide sequence analysis of antibiotic resistance genes in bacterial strains is controversial [68, 69]. As a result, the regulatory authorities still need to conduct complete toxicological detection screens and strain identification of this bacterium.

#### 2.3.3. Bacteroides fragilis

*Bacteroides fragilis* belong to Bacteroides and are long, rod-shaped, gram-negative bacteria. *B. fragilis* can be transmitted from mother to child during vaginal delivery; thus, it becomes the main inhabitant of the intestinal tract, and accounts for approximately 25% of the total number of intestinal bacteria [70]. *B. fragilis* is responsible for IBD [71], pathogenic bacterial infection, [72], and tumor immunity [73] in humans. Further, *B. fragilis* can activate the adaptive immune system of the host by binding to immunoglobin A (IgA) using sugar molecules on their surface to stabilize the colonization in the colonic epithelial mucosa [74, 75]. They promote the maturation of human immune system, inhibit inflammation, adjust the structure of intestinal flora, and are believed to be beneficial for human health. Therefore, *B. fragilis* have been suggested as a candidate probiotic because of their ability to inhibit pathogenic bacteria [76].

In the colon, *B. fragilis* metabolizes a variety of carbohydrates and secretes eight different types of capsular polysaccharides, one of which is polysaccharide A (PSA), a unique amphoteric polysaccharide and an immune regulator. PSA can be internalized and processed by antigen presenting cells (with MHC class II molecules) for recognition by T cells [77], suggesting continuous boosting of *the* host *immune system* with PSA [78]. In human peripheral blood mononuclear cells, PSA promotes the differentiation of initial T cells into T_reg_ cells by regulating dendritic cell, induces the expression of Foxp3 and CD39, inhibits the inflammatory factor IL-17, and promotes the secretion of IL-10 [79], which reportedly helps treat intestinal inflammation. In a viral meningitis mice model, researchers have observed that PSA can prevent death from a lethal herpes simplex virus infection. PSA induces the secretion of IL-10-specific T and B cells to limit the inflammatory response in the brain and reduce the mortality in mice [80]. However, there is considerable specificity between strains. Chan et al. [81] have determined that defective *B. fragilis* NCTC 9343 have IL-10, IL-17, and IFN-*γ* immune responses similar to those induced by PSA in colitis and tumors, and this effect is not dependent on PSA. Hence, treatment with *B. fragilis* or purified PSA is a novel therapeutic approach for human autoimmune diseases and IBD.

*B. fragilis* Zy-312 is an NGP candidate that is isolated from the intestinal tract of healthy infants [82] and can treat antibiotic-induced diarrhea [82], intestinal infection caused by *Vibrio parahaemolyticus* [76], and *Enterobacter sakazakii*-induced enteritis by regulating the intestinal flora [83]. In fact, it is expected to become a living biological drug. In 2017, Wang et al. [84] isolated and identified different strains of *B. fragilis*, evaluated their safety, and determined their pharmacology, and other microbiome-related research. Their results showed that *B. fragilis* Zy-312 is safe in normal and immunodeficient mice. Further, whole-genome sequencing showed that *BFT* (an enterotoxin gene) is missing in this strain, and 33 virus-related factors mainly encode structural proteins and proteins with physiological activity. The bacterium is resistant to cefepime, kanamycin, and streptomycin; however, there are no mobile drug resistant elements that rule out the risk of plasmid-mediated antibiotic resistance transfer. Notably, *B. fragilis* Zy-312 shows high genetic stability after 100 generations in vitro [84]. Preclinical study using *B. fragilis* as a viable drug has shown considerable efficacy and safety, thereby providing a new therapeutic option for patients with ulcerative colitis (UC). SK08 live bacterial powder, a viable drug developed using *B. fragilis*, has been approved by the Chinese Food and Drug Administration and is currently in the clinical trial stage. The drug is registered and classified as a therapeutic biological product [85]. The safety and efficacy of SK08 in patients with irritable bowel syndrome. http://www.chinadrugtrials.org.cn/clinicaltrials.searchlist.dhtml. Accessed 5 July 2021).

#### 2.3.4. Eubacterium hallii

Another potential candidate for future probiotic formulations, *E. hallii*, is a gram-positive bacterium that belongs to the phylum Firmicutes and has an abundance of approximately 3% in an adult human [86]. Different carbon sources can be widely used, including sugars and organic acids [87]. Short chain fatty acid (SCFA) plays crucial roles in intestinal tract health, which stimulate the production and differentiation of enterocytes, improving mucus production and epithelial health. Missing SCFA will trigger an inflammatory response [88].

In the early stages of life, *E. hallii* interacts with *Bifidobacterium infantile* to degrade intermediates of breast milk oligosaccharides to produce SCFAs [89]. Further, the abundance of *E. hallii* in patients with UC is significantly lower compared with healthy controls [90]. After transplantation of fecal bacteria, the abundance of *E. hallii* increases in patients with UC due to intestinal SCFA synthesis and increase in secondary bile acid levels; consequently, this eases the symptoms in human intestinal inflammation [91]. Udayappan et al. [92] have assessed the role of *E. hallii* in obesity and diabetes mice models. The results show that *E. hallii* metabolize butyric acid to activate G-coupling protein receptor signaling pathway, improve GLP1 and GLP2 production, do not affect body weight or food intake, strengthen the intestinal barrier function, and improve insulin sensitivity and energy metabolism. These results show that the strain could be safely used and be effective against insulin sensitivity. PhIP(2-Amino-1-methyl-6-phenylimidazo[4,5-b]pyridine) has been considered a mutagen and carcinogen in the colon. Many findings further indicate the high exposure of *PhIP* in human diet regardless of country of origin [93]. Notably, Fekry et al. [94] have found that *E. hallii* derived converted *PhIP* into *PhIP*-*M1* ([7- hydroxy-5-methyl-3-phenyl-6,7,8,9-tetrahydropyrido [3',2':4,5] imidazo [1,2-a]pyrimidin-5-ium chloride). Further, *E. hallii* has a positive effect on the metabolism of specific compounds, decomposes toxic substance, and protects the colon.

Currently, Caelus Health is working with a Danish company, Korhansson, to develop oral preparations containing *E. hallii* as biological therapeutic agents to reduce insulin resistance and prevent the development of T2D in patients with metabolic syndrome (ClinicalTrials.gov, 2020. Efficacy and safety of 12-weeks supplementation of *E. hallii* on insulin sensitivity and glycaemic control. efficacy and safety of 12-weeks supplementation of *E. halli*i on insulin sensitivity and glycaemic control-Full Text View-[95].

#### 2.3.5. *Roseburia* spp.

*Roseburia* spp. is a butyrate-producing, gram-positive, obligate anaerobe with a slightly curved rod-like structure. It belongs to the *Clostridium* XIVa subgroup [96], and its abundance in the intestinal tract of a healthy person is approximately 3–15% [97]. Further, it is associated with inflammation resistance [98], Parkinson's disease [99], and IBD [100], and is considered as a candidate NGP. People with cardiovascular disease have lower levels of *Roseburia* and other butyrate-producing bacteria. Interestingly, atherosclerosis was reduced in mice that were fed both *Roseburia* and a high-fiber diet, wherein the high-fiber diet mediated the production of butyrate from *Roseburia* to reduce atherosclerosis [101].

Current prospective studies on flagellin, an important structural component of the bacterial flagellum, have focused on the association between receptors and intestinal inflammation. In a recent study with 212 participants, the effects of alcohol intake on gut flora were analyzed by excluding host genes and other potential confounding factors. The results showed that alcohol intake was associated with reduced *Roseburia* spp. abundance, After oral administration of *Roseburia intestinalis*, flagellin upregulated the tight junctions of *Occludin*, *MUC2*, and *REG3* through TLR5, thereby restoring the integrity of intestinal epithelial cells of mice with alcoholic fatty liver disease and preventing the possible occurrence of intestinal fistula [102]. Similarly, Wu et al. [103] have observed in dextran sulphate sodium-induced colitis model of C57BL/6 mice and LPS/ATP-induced THP-1 macrophage model that *R. intestinalis* (R.I.) flagellin inhibits inflammatory body-induced apoptosis and alleviates intestinal inflammation through mir-223-3p/NLRP3 signal transduction. Therefore, R.I. flagellin reportedly has a protective role during treatment of alcoholic fatty liver and improves UC.

However, whether the response of gut microbe intervention is beneficial depends on age and health. This novel finding suggests that *R. intestinalis* can trigger antiphospholipid syndrome, particularly in genetically susceptible individuals [104]. Therefore, *R. intestinalis* can cause an autoimmune disease in susceptible mice. As clinical data on *Roseburia* are still in infancy, it is challenging to define the exact role (beneficial or not) of the strain. Thus, it is necessary to develop and establish accurate and meaningful clinical models to further elucidate the relationship of *Roseburia* with human diseases.

### 2.4. Isolation of NGPs

There are significant differences between the way traditional probiotics and NGPs studied, driven by the current high-throughput technologies available and cumulated data evidence (Table 1). Discovery of traditional probiotics is attributed to a top-down screening strategy, that is, screening of microbes that are enriched in healthy individuals compared with those in diseased individuals. The “experience-first” method guides the development of probiotics; however, because of absence of information on the underlying mechanisms, this method must rely on repeated experiments and complex screening processes to determine the exact health benefits [28].

Like drug development, the NGP screening strategy can follow two bottom-up development approaches: phenotypic-based and target-based. Phenotypic methods are screened according to the effects of cells and animals on specific strains using in vitro cultures, cells, and animal models of specific diseases. The strategy of discovering NGP candidates based on a target mainly depends on sequencing to evaluate and predict the ability of the bacteria themselves or their metabolites to produce molecular effectors that can regulate host or microbial-related signaling pathways. Studies based on these aspects will help resolve the function, safety, and molecular mechanism of NGPs in animals. Due to the difficulty in replicating essential aspects of their anaerobic environment, some bacteria species cannot be cultured and isolated yet. However, new media and modified procedures, such as improved culturomics, are constantly developing and evolving. They are composed of a variety of culture conditions with rapid identification of bacteria, raising the level of cultured bacteria and their possible use as bioresources or even NGP.  Table 2 summarizes the main putative new species in recent culturing methods. The US FDA has launched a “Live Biotherapeutic Products” (LBP) program to specifically regulate the application, clinical trials, and commercialization of emerging probiotics. It is expected that soon NGPs will become a reality in the healthcare industry.

### 2.5. Translation to Clinical Applications

Future microbiome studies should focus on clinical translational research. Currently, there is slow progress with respect to the therapeutic effects of FMT in recurrent *C. difficile*-related diseases. Development of NGPs has accelerated the research into human pathology and physiology. For specific diseases, targeted, synthesizing, and living biological drugs composed of candidate strains of NGPs can be a new microbial therapy instead of FMT. Living biological drugs have three advantages: (1) the drug is known and controllable, and the drug composition is more accurate in comparison with FMT; (2) types of bacteria in the drug composition are repetitive; and (3) the FDA and the European Pharmacopoeia Commission have clearly defined LBPs, which makes their safety regulation easier.

In 2020, Seres announced the results of a phase III clinical trial of an intestinal microbiome drug, SER-109, that can effectively reduce the risk of recurrence of CDI [105]. Seres therapeutics announces U.S. Food and Drug Administration correspondence following positive ser-109 phase 3 study results. https://ir.serestherapeutics.com/news-releases/news-release-details/seres-therapeutics-announces-us-food-and-drug-administration. Accessed 5 July 2021). In May 2020, Rebiotix announced that the intestinal microbiome drug, RBX2660, also targets CDI, and has made positive progress in phase III clinical trials [106]. Rebiotix and Ferring announce the world's first with positive preliminary pivotal phase 3 data for investigational microbiome-based therapy RBX2660. https://www.rebiotix.com/news-media/press-releases/rebiotix-announces-worlds-first-positive-pivotal-phase-3-data-investigational-microbiome-based-therapy-rbx2660/. Accessed 5 July 2021). In October 2020, 4D Pharma announced positive progress in phase I/II clinical trials from its phase II BHT-II-002 trial of Blautix®, a single strain live biotherapeutic to treat IBS [107]. 4D Pharma announces topline results from Blautix® phase II trial in irritable bowel syndrome (IBS). https://www.4dpharmaplc.com/en/newsroom/press-releases/4d-pharma-announces-topline-results-blautix-phase-ii-trial-ibs. Accessed 5 July 2021).

## 3. Conclusion and Perspectives

To summarize, NGPs are a marked development over traditional probiotics, which have a history of safe use in humans. *Akkermansia muciniphila*, *Faecalibacterium prausnitzii*, *Bacteroides fragilis*, *Eubacterium hallii*, and *Roseburia* spp. have shown great potential as NGPs for disease intervention and treatment. Specific strains also have positive effects on human gastrointestinal, metabolic, and immune system diseases. At present, many research institutions, pharmaceuticals, and food enterprises are focused on developing NGPs. However, because of the uniqueness of NGPs, they are more suitable in biopharmaceuticals. The human body and microorganisms form a complex ecosystem, and it is very challenging to determine a correlation between individual strains and their hosts. Thus, in future, NGPs will have to overcome obstacles such as personalization and interaction within flora. Hence, thorough in-depth research is still required to authenticate their safety and effectiveness.

## Figures and Tables

**Table 1:** Some important points in the assessment of traditional probiotics and NGPs.

Traditional probiotics	NGPs
Mostly derived from gut, breast milk, and fermented foods; anaerobe	Identified from comparison results between healthy volunteer; obligately anaerobe
MRS, M17, YPD is a commonly used rich medium for traditional probiotics cultivation	The culture media most used for cultivation of NGPs with success were new media and modified procedures
Usually considered safe at the strain level by the US FDA, or qualified safety hypothesis (QPS) at the species level by EFSA	Strict security and regulations are needed. Their beneficial properties, antibiotic resistance profiling, history of safe use (if available), publication of the genomic sequence, toxicological studies in agreement with novel food regulations, and the qualified presumptions of safety should be evaluated
For general subhealthy people	The function of individual strains may be different for specific diseases
As food or food supplements	Included in LBP's food additive category

**Table 2:** Culturing approaches to NGPs and Candidatus species.

Reference	Bacteria species/strain	Culture media	Disease target
[39]	*Akkermansia muciniphila* ATCCBAA-835	BHI, soft agar mucin medium	Type 2 diabetes and weight controls
[61]	*Faecalibacterium prausnitzii* ATCC 27766	YCFA	Chronic moderate and severe colitis
[83]	*Bacteroides fragilis* ZY-312	BHI	Necrotizing enterocolitis
[108]	*Eubacterium hallii* DSM 3353	YCFA and contained approximately 33 mM acetate	*Balance* of intestinal microflora
[109]	*Roseburia intestinalis* DSMZ-14610	Composition of medium: processed water 40 ml, soybean-casein, digest broth 2.75%, yeast extract 0.2%, animal tissue digest 0.05%, dextrose 0.2%, hemin 0.0005%, menadione 0.00005%, sodium citrate 0.02%, thiols 0.1%, sodium pyruvate 0.1%, saponin 0.26%, antifoaming agent 0.01%, sodium polyanetholsulfonate 0.035%	Crohn's disease

YCFA: yeast-extract-casein hydrolysate-fatty acids; BHI: brain–heart infusion.

## Abbreviations

NGPs: Next-generation probiotics
FMT: Fecal microbiota transplantation
IBD: Inflammatory bowel disease
IBS: Irritable bowel syndrome.
